# Regulation of the intestinal barrier by nutrients: The role of tight junctions

**DOI:** 10.1111/asj.13357

**Published:** 2020-03-17

**Authors:** Takuya Suzuki

**Affiliations:** ^1^ Department of Biofunctional Science and Technology Graduate School of Biosphere Science Hiroshima University Higashi‐Hiroshima Japan; ^2^ Program of Food and AgriLife Science Graduate School of Integrated Sciences for Life Hiroshima University Higashi‐Hiroshima Japan

**Keywords:** intestinal barrier, intestinal permeability, nutrient, tight junction

## Abstract

Tight junctions (TJs) play an important role in intestinal barrier function. TJs in intestinal epithelial cells are composed of different junctional molecules, such as claudin and occludin, and regulate the paracellular permeability of water, ions, and macromolecules in adjacent cells. One of the most important roles of the TJ structure is to provide a physical barrier to luminal inflammatory molecules. Impaired integrity and structure of the TJ barrier result in a forcible activation of immune cells and chronic inflammation in different tissues. According to recent studies, the intestinal TJ barrier could be regulated, as a potential target, by dietary factors to prevent and reduce different inflammatory disorders, although the precise mechanisms underlying the dietary regulation remain unclear. This review summarizes currently available information on the regulation of the intestinal TJ barrier by food components.

## 1 INTRODUCTION

One of the most important roles of the intestinal epithelium is to digest ingested food and to absorb nutrients and dietary factors. The epithelium provides a biochemical and physical barrier to the diffusion of pathogens, toxins, and allergens from the lumen to the mucosal tissues (Peterson & Artis, 2014). Defects in barrier integrity robustly activate immune cells and cause chronic inflammation of the intestinal tissues. When the barrier is impaired, inflammatory molecules, such as endotoxins, can reach the different organs via the circulation and play a role in the pathogenesis of non‐intestinal disorders, such as alcoholic and non‐alcoholic liver diseases, diabetes, obesity, and chronic kidney disease.

**FIGURE 1:**
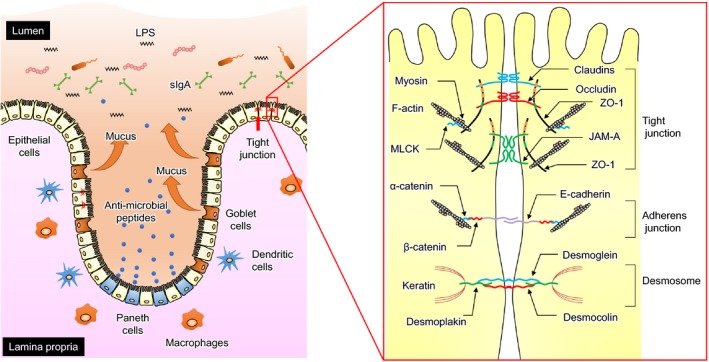
Anatomy of the intestinal barrier. Intestinal epithelial cells constitute a biochemical and physical barrier to the diffusion of pathogens, toxins, and allergens from the intestinal lumen to the mucosal tissues (left panel). The intestinal barrier system depends on interactions among several barrier components, including the adhesive mucous gel layer, immunoglobulin A, antimicrobial peptides, and intercellular tight junctions (TJs). TJs are formed by a multiple‐protein complex located in the apical portion of the lateral membrane of epithelial cells (right panel). The TJ structure comprises transmembrane proteins, such as claudin, occludin, and junctional adhesion molecule‐A (JAM‐A), and intracellular plaque proteins, such as zonula occludens (ZOs) and cingulin. The interaction between extracellular regions of the transmembrane proteins in adjacent cells regulates the paracellular passage of molecules

The intestinal barrier system depends on interactions among several barrier components, including the adhesive mucous gel layer, immunoglobulin A, antibacterial peptides, and intercellular tight junctions (TJs; Figure 1). Among these components, the TJs constitute the major determinant of the intestinal physical barrier. TJs are formed by the assembly of a multiple proteins located close at the apical portion of the lateral membrane of epithelial cells. The TJ structure consists of transmembrane proteins, such as claudin (Furuse, Fujita, Hiiragi, Fujimoto, & Tsukita, 1998), occludin (Furuse et al., 1993), tricellulin (Ikenouchi, Furuse, Furuse, Sasaki, & Tsukita, 2005), and junctional adhesion molecule‐A (JAM‐A) (Martin‐Padura et al., 1998), and intracellular plaque proteins, such as zonula occludens (ZO) and cingulin (Citi, Sabanay, Jakes, Geiger, & Kendrick‐Jones, 1988). The interactions between extracellular regions of the transmembrane proteins of adjacent cells regulate the paracellular passage of molecules. TJ pathways can be roughly categorized into bicellular and tricellular junctions (Ikenouchi et al., 2005). The bicellular TJ strands are formed by two adjacent epithelial cells, whereas the tricellular TJ strand is formed at the meeting point of three cells, where three TJ strands converge.

Claudins are encoded by a multigene family composed of at least 24 members. Each protein shows a unique tissue expression pattern. Claudin‐2, ‐3, ‐4, ‐7, ‐12, ‐14, and ‐15 isoforms are relatively abundant in the epithelium of the small and large intestines (Holmes, Itallie, Rasmussen, & Anderson, 2006). These proteins are functionally divided into two types: barrier‐forming isoforms and pore‐forming isoforms. Claudins 3, 4, 7, and 14 form a selective barrier to macromolecules and ions, whereas claudins 2, 12, and 15 form selective pores to ions and water and thereby increase the intestinal permeability (Van Itallie & Anderson, 2006).

The intracellular domains of transmembrane proteins, such as claudins and occludin, are associated with cytosolic plaque proteins, which provide a structural scaffold to the TJ. The scaffold molecules in a TJ include ZOs and Cinglin. ZO‐1, ‐2, and ‐3 belong to membrane‐associated guanylate kinase homologs (MAGUKs), which contain multidomain structures, such as 3 PDZ domain, a Src homology‐3 (SH3), and an enzymatically inactive GUK domain, in their N‐terminal region (Gonzalez‐Mariscal, Betanzos, Nava, & Jaramillo, 2003). Occludin and many claudin isoforms bind to these domains, whereas the C‐terminal regions of ZOs interact with the actin cytoskeleton (Furuse et al., 1994; Haskins, Gu, Wittchen, Hibbard, & Stevenson, 1998; Itoh et al., 1999). The association of the cytoskeleton with the TJ structure seems to be essential for the regulation and maintenance of TJ function (Fanning, Ma, & Anderson, 2002). In addition, the interaction between ZO proteins is achieved through a second PDZ domain (Gumbiner, Lowenkopf, & Apatira, 1991; Haskins et al., 1998). Although the cellular and biochemical functions of ZOs are still under investigation, ZOs are known to regulate the assembly as well as stabilization of TJ structure. Epithelial Eph4 cells express ZO‐1 and ‐2 but not ZO‐3. Knockout of ZO‐1 in Eph4 cells induces an obvious delay in the incorporation of TJ molecules, including occludin and claudins, into the TJ structure (Umeda et al., 2004). In addition, the TJ structure was mostly lacking, and no claudins were observed at the cell borders when ZO‐2 expression was suppressed by RNAi technology in ZO‐1‐deficient Eph4 cells.

As described above, impairment of the TJ barrier in the intestine results in systemic and intestinal inflammation and plays an important role in the pathogenesis of various disorders. Therefore, the maintenance and protection of the TJ barrier using nutrition and dietary factors could be effective for preventing diseases. The current review summarizes findings regarding the regulation of TJ barrier and paracellular permeability by dietary factors. The studies described are primarily related to human physiology. However, many findings could be translated to general animal physiology because nutrition and dietary factors are often shared between humans and animals. Knowledge about TJ barrier regulation by single dietary factors is limited. Much more information is available on the effect of commensal and probiotic bacteria on the regulation of the intestinal barrier. These aspects are not extensively discussed in the current review as they have been recently reviewed elsewhere (Bron et al., 2017; Hiippala et al., 2018).

## 2 CARBOHYDRATES AND SUGARS

Carbohydrates and sugars are one of the main energy sources and usually constitute the highest percentage of dietary nutrients. However, their roles in the regulation of intestinal barrier have not been delineated to date. The effects of high‐glucose and high‐fructose diets on the intestinal barrier have been investigated in mice because diabetes and obesity are closely associated with endotoxemia and systemic inflammation. Feeding mouse high‐glucose and high‐fructose diets (65% of calories are obtained from carbohydrates; of those, 85% come from glucose or fructose, and 15% from sucrose) for 12 weeks induces endotoxemia and increases the intestinal permeability to fluorescein isothiocyanate (FITC)‐labeled dextran, a marker of epithelial TJ function (Do, Lee, Oh, Kim, & Park, 2018). The diets result in decreased levels of ZO‐1 and occludin, and increased levels of inflammatory cytokines, such as tumor necrosis factor (TNF)‐α and interleukin (IL)‐1β, in the colon. Precise mechanisms underlying the impairment of TJ barrier are not known, but the dysbiosis of intestinal microflora induced by high‐glucose and high‐fructose diets appears to play a role (Do et al., 2018). Furthermore, luminal glucose increases the intestinal permeability to small‐sized nutrient molecules by activating myosin light‐chain kinase (MLCK; Turner et al., 1997). The regulation of TJ permeability appears to be physiologically associated with sodium‐dependent glucose transporter‐1 (SGLT‐1). Transporter activation in the apical membrane of the epithelial cell activates MLCK and increases MLC phosphorylation. The contraction of the perijunctional actomyosin ring induced by MLC phosphorylation results in increased paracellular permeability.

## 3 PROTEINS, PEPTIDES, AND AMINO ACIDS

Proteins are essential for the growth and repair of the body and maintenance of good health and are involved in a wide range of metabolic interactions. However, the role of proteins in intestinal barrier regulation has not been extensively investigated to date. Of note, however, is that some dietary peptides produced by the enzymatic digestion of dietary proteins reportedly enhance or protect the intestinal barrier and may be potentially used to ease intestinal diseases. Furthermore, amino acids, such as glutamine and tryptophan, play protective roles in the intestinal barrier. This section summarizes intestinal TJ regulation by dietary proteins, peptides, and amino acids.

### 3.1 Protein nutrition

Zhu, Shi, Niu, Wang, and Zhu (2018) examined the TJ proteins in the jejunum of rats fed diets with different protein content for 2, 4, and 10 weeks. The outcomes were complex. Feeding low‐protein diets (12% wt/wt) for 2 weeks decreased the ZO‐1 and occludin expression in the jejunum, but not in the colon, compared with rats fed a control protein diet (19.3% wt/wt). On the other hand, the ZO‐1 and occludin levels after 4 weeks, and occludin levels after 10 weeks, in the jejunum and colon of rats fed the low‐protein diet were higher than those in rats fed the normal diet. Although the precise mechanisms underlying these observations are still unclear, alteration of the intestinal microflora by dietary protein restriction may have been involved in the observed effects.

### 3.2 Peptides

Several bioactive peptides are prepared from protein‐rich foods, such as milk, egg, and soybean for dietary supplements. Peptides prepared from milk, soy, and Alaska pollock can potentially regulate intestinal barrier function. However, dietary intervention involving these peptides in animals or humans has not yet been explored.

The pentapeptide NPWDQ derived from bovine α_s‐2_ casein reinforces the barrier integrity of intestinal Caco‐2 cells, leading to the inhibition of ovalbumin permeation (Tanabe et al., 2006). This peptide was originally isolated from an enzymatic hydrolysate of cheese. Upregulation of occludin has been suggested as the underlying mechanism (Yasumatsu & Tanabe, 2010).

Tryptic peptides from collagen derived from the skin of Alaska pollock reduce TNF‐α–induced disruption of the TJ barrier in Caco‐2 cells (Chen et al., 2017). Although the primary structure of the peptide(s) has not yet been delineated, the peptide(s) attenuates the decrease in occludin and ZO‐1 levels, as well as the increase in MLC phosphorylation by inhibiting nuclear factor‐kappa B (NF‐κB) and extracellular signal‐regulated kinase (ERK) pathways.

The enzymatic hydrolysate of β‐conglycinin, a major soybean protein, reinforces the barrier integrity of intestinal Caco‐2 cells (Yang et al., 2008). The molecular weight of the bioactive peptide is predicted to be 10–20 kDa. The peptide inhibits the translocation of *Salmonella typhimurium* by increasing the transepithelial electrical resistance (TER), a marker of TJ function. On the other hand, intact β‐conglycinin increases the TJ permeability of porcine intestinal IPEC‐J2 cells (Peng et al., 2019). The β‐conglycinin–mediated disruption of TJ structure and cytoskeleton appears to be linked to the activation of inducible nitric oxide synthase, NF‐κB, c‐Jun N‐terminal kinases (JNK), and p38 mitogen‐activated protein kinase (MAPK).

### 3.3 Amino acids

Glutamine is the most abundant free amino acid in the human body and is a major substrate of intestinal cells. Accumulating evidence from basic and clinical studies suggests that it plays important roles in the maintenance and protection of the intestinal barrier. In clinical studies, the lactulose/mannitol ratio in the urine following oral administration is often evaluated as a test of intestinal permeability because the method is non‐invasive. Glutamine supplementation reduces the intestinal hyperpermeability in healthy and malnourished children and in individuals with irritable bowel syndrome (IBS), Crohn's disease, and severe burns, as indicated by a lower lactulose/mannitol ratio in these individuals than that in the control individuals (Benjamin et al., 2012; Lima et al., 2005, 2007; Peng, Yan, You, Wang, & Wang, 2004; Zhou et al., 2019). For example, in individuals with postinfectious, diarrheal‐predominant, irritable bowel syndrome (IBS‐D), glutamine supplementation (5 g/day) reduces intestinal hyperpermeability and also improves the clinical score, bowel movement frequency, and stool condition (Zhou et al., 2019). In a separate study, colonic biopsies of individuals with IBS‐D were incubated with 0.6 and 10 mmol/L glutamine; claudin‐1 expression in the 10 mmol/L group was higher than that in the 0.6 mmol/L group (Bertrand et al., 2016). Protective effects of glutamine on the intestinal TJ structure were also demonstrated in rodent models of different diseases, such as alcoholic liver disease, IBS, chemotherapy, inflammatory bowel diseases, graft‐versus‐host disease, and infection (Beutheu et al., 2014; Chaudhry et al., 2016; Ewaschuk, Murdoch, Johnson, Madsen, & Field, 2011; Noth et al., 2013). Molecular mechanisms of the intestinal barrier defect in these in vivo models vary and are complex.

Several studies relied on cell culture approaches to examine the maintenance, promotion, and protection of the intestinal TJ structure by glutamine. Accordingly, glutamine depletion induced by glutamine‐free media and glutamine synthase inhibition increases the intestinal permeability and reduces ZO‐1, occludin, and claudin‐1 levels in the detergent‐insoluble fraction of Caco‐2 cells (Li & Neu, 2009). The phosphatidylinositol 3‐kinase/Akt pathway might be involved in TJ disruption. Similarly, glutamine supplementation increases claudin‐1 expression, but not ZO‐1 or occludin expression, in Caco‐2 cells (Li, Lewis, Samuelson, Liboni, & Neu, 2004). In porcine intestinal porcine epithelial cells (IPEC)‐1, glutamine enhances the barrier integrity and TJ protein levels, e.g., those of ZO, occludin, claudins, and JAM‐A, via the calcium/calmodulin‐dependent kinase 2‐AMP–activated protein kinase (AMPK) pathway (Wang et al., 2016). In addition, glutamine may potentially prevent TJ disruption induced by methotrexate and acetaldehyde, although the underlying mechanisms seem to be unrelated. Glutamine protects the TJ structure against methotrexate by suppressing the JNK, ERK1/2, and NF‐κB pathways, whereas the protective effect against acetaldehyde is mediated by activation of EGF receptor tyrosine kinase (Beutheu Youmba et al., 2012; Seth, Basuroy, Sheth, & Rao, 2004).

Another amino acid, tryptophan, also supports the intestinal TJ barrier; however, excessive dietary levels of tryptophan may have an adverse effect. In the murine model of non‐alcoholic fatty liver disease (NAFLD), tryptophan supplementation (0.24% wt/wt) reversed the occludin expression in the jejunum and improved the disease‐related liver parameters (Ritze, Bardos, Hubert, Bohle, & Bischoff, 2014). Feeding weaned pigs a 0.2% and 0.4% tryptophan diet for 4 weeks increases the ZO‐1, ZO‐3, and claudin‐3 levels in the jejunum (Liang et al., 2018). Similar results were obtained with a 0.4% tryptophan diet in finishing pigs (Liu et al., 2017). However, while feeding the weaned pig 0.75% tryptophan diet for 3 weeks decreases the occludin and ZO‐1 expression at the mRNA level, an increasing trend of these mRNA levels is observed in animals fed 0.15% tryptophan diet (Tossou et al., 2016). In an in vitro study using Caco‐2 cells, tryptophan supplementation alleviated lipopolysaccharide (LPS)‐induced injury of the TJ barrier when administered at a dose of 40 μM but not 80 μM (Chen et al., 2019). The lower dose of tryptophan reversed the LPS‐induced upregulation of MLCK and downregulation of claudin‐1, but not ZO‐1 or occludin levels. In addition, tryptophan upregulates TJ protein levels, such as occludin, claudin‐4, and ZO‐1 levels, but not claudin‐1 or ZO‐3 levels, by activating mammalian target of rapamycin in IPEC‐1 cells (Wang et al., 2015).

## 4 LIPIDS

A growing body of evidence demonstrates that the intake of high amounts of fat induces intestinal hyperpermeability, which plays a pivotal role in the pathogenesis of metabolic disorders. In addition, some fatty acids influence intestinal TJ barrier regulation in a chain length‐ and structure‐dependent manner. This section summarizes effects of high fat diets, long‐chain fatty acids (LCFA), and medium‐chain fatty acids (MCFA) on the intestinal TJ barrier. The effects of short‐chain fatty acids (SCFAs), such as acetic, propionic, and butyric acids, on the intestinal barrier are described in Section 6.

### 4.1 High‐fat diet

Metabolic endotoxemia resulting in low‐grade systemic inflammation plays an important role in the pathogenesis of obesity, type 2 diabetes, and metabolic syndrome. LPS enters the circulation either after incorporation into bile acid micelles or by paracellular diffusion in the intestinal epithelium (Moreira, Texeira, Ferreira, Peluzio Mdo, & Alfenas Rde, 2012). The former is independent of intestinal barrier disruption. LPS is incorporated into micelles via its insoluble lipid A structure and is then absorbed by epithelial cells and aggregated into the chylomicrons during the postprandial period (Ghoshal, Witta, Zhong, Villiers, & Eckhardt, 2009). The latter seems to be underpinned by a different mechanism, during the feeding of high‐fat diets.

High‐fat diet impairs the TJ structure and induces hyperpermeability in the small and large intestines of rodents. Activation of mucosal immune cells, including lymphocytes and mast cells, by absorbed fat seems to be one of the underlying mechanisms of the hyperpermeability (Ji, Sakata, & Tso, 2011). LCFAs derived from dietary fats activate the immune cells, which release a wide variety of inflammatory mediators, including interferon‐γ, TNF‐α, IL‐1β, IL‐6, and proteases. These mediators impair the TJ barrier by downregulating TJ protein levels and upregulating MLCK (Al‐Sadi & Ma, 2007; Suzuki, Yoshinaga, & Tanabe, 2011; Wang et al., 2005). In addition, excessive bile acid secretion, which is induced by high‐fat feeding, negatively regulates the intestinal TJ barrier. The author of the current review and colleagues demonstrated a positive correlation between the intestinal permeability to FITC‐labeled dextran and the cecal concentration of bile acids in rats fed a high‐fat diet (Murakami, Tanabe, & Suzuki, 2016). Furthermore, TJ protein expression in Caco‐2 cells exposed to pathological concentrations of rat bile juice, which contains different bile acids, is lower than that in the control cells (Suzuki & Hara, 2010).

Impairment of TJ barrier by individual bile acids has been also examined in Caco‐2 cells, although the underlying mechanisms remain controversial. Raimondi et al. (2008) demonstrated that chenodeoxycholic and deoxycholic acids induce occludin redistribution by activating the EGF receptor. Araki et al. (2005) suggested that cholic acid‐induced hyperpermeability occurs as a consequence of reactive oxygen production. Furthermore, alterations in the composition of the intestinal microflora may also be associated with the impairment of TJ barrier by high‐fat diets. Although the mechanism is likely complex, reduction in *Bifidobacterium* spp. abundance may at least partly play a role in the TJ barrier impairment. Indeed, plasma LPS levels are negatively correlated with cecal *Bifidobacterium* spp. levels in mouse fed high‐fat diet and an increase in bifidobacterial abundance by oligofructose reduces endotoxemia (Cani et al., 2007).

### 4.2 Fatty acids

LCFAs are major components of dietary fat and play essential roles in cellular function, including acting as cell membrane components and energy sources and involvement in eicosanoid synthesis. The physiological regulation of TJ permeability by individual LCFAs, such as arachidonic, linoleic, eicosapentaenoic, docosahexaenoic, and γ‐linolenic acids (AA, LA, EPA, DHA, and GLA, respectively), has been investigated, although some observations are controversial. In Caco‐2 cells, under normal conditions (i.e., without any disruption of the intestinal barrier), LA, EPA, DHA, and GLA appear to increase TJ permeability (Usami, Komurasaki, Hanada, Kinoshita, & Ohata, 2003; Usami et al., 2001). The EPA‐ and DHA‐induced hyperpermeability might be caused by the formation of eicosanoids. Prostaglandin E3, which is derived from *n*‐3 fatty acids, such as EPA and DHA, increases TJ permeability by redistributing occludin and claudin‐4 (Rodriguez‐Lagunas, Ferrer, & Moreno, 2013). However, γ‐LA, AA, EPA, and DHA decrease the TJ permeability of intestinal T84 cells (Willemsen et al., 2008). Some LCFAs reportedly protect the barrier integrity against noxious stimuli. Heat stress impairs TJ barrier and structure in Caco‐2 cells, but EPA and DHA reduce hyperpermeability and restore the expression of occludin and/or ZO‐1 in these cells under heat stress (Xiao et al., 2013). In addition, EPA, DHA, and AA suppress IL‐4–induced hyperpermeability of T84 cells (Willemsen et al., 2008).

Medium‐chain fatty acids are present in milk fat, palm oil, and palm kernel oil. Caprylic (C8), capric (C10), and lauric (C12) acids have been investigated as enhancers of drug absorption via the TJ pathway (Lindmark, Nikkila, & Artursson, 1995). The underlying mechanisms of C10‐ and C12‐mediated enhancement of TJ permeability have been examined (Lindmark, Kimura, & Artursson, 1998; Lindmark, Schipper, Lazorova, de Boer, & Artursson, 1998; Tomita, Hayashi, & Awazu, 1996). In Caco‐2 cells, C10 and C12 enhance TJ permeability by activating protein kinase C (PKC) and MLCK. MLCK induces the contraction of the perijunctional actomyosin ring, resulting in increased paracellular permeability. The C10 effect also involves phospholipase C activation and ZO‐1 redistribution. C10‐induced TJ permeability was also demonstrated in the intestines of rats and humans (Shimazaki, Tomita, Sadahiro, Hayashi, & Awazu, 1998; Soderholm et al., 1998). Interestingly, fluorescent visualization using sulpho‐NHS‐SS‐biotin revealed that C10 and C12 predominantly enhance the tricellular and bicellular pathways, respectively (Dittmann et al., 2014; Krug et al., 2013).

## 5 MINERALS

Minerals are inorganic substances present in all body tissues and fluids and necessary for the maintenance of certain essential biological processes. In this section, the effects of zinc on the intestinal TJ barrier are described.

Among minerals, the promotive and protective roles of zinc in the intestinal barrier have been investigated in studies involving animals and cell cultures. Indeed, zinc supplementation reduces the intestinal barrier defect caused by malnutrition, colitis, and infection (Rodriguez et al., 1996; Sturniolo et al., 2002). Although different mechanisms may be involved in the zinc‐mediated protection of the intestinal TJ barrier, a zinc sensing receptor, GPR39, which senses the extracellular zinc, is involved in barrier regulation. In *Gpr39* knockout (KO) mice, the expression of occludin and ZO‐1 is reduced compared with that in the wild‐type mouse (Cohen, Sekler, & Hershfinkel, 2014). Furthermore, the absence of GPR39 results in enhanced susceptibility to dextran sulfate sodium (DSS)‐induced colitis, as indicated by more severe disease symptoms and tissue destruction than those observed in the wild‐type mouse (Sunuwar, Medini, Cohen, Sekler, & Hershfinkel, 2016). Furthermore, the recovery of colonic inflammation and TJ barrier following the removal of DSS is slower in the *Gpr39* KO mouse than in the wild‐type mouse.

Molecular mechanisms underlying GPR39‐mediated regulation of the TJ barrier were also examined in cell culture. In Caco‐2 cells, upregulation of PKCζ after GPR39 activation by zinc inhibits the decrease in occludin and ZO‐1 levels caused by *Salmonella enterica* serovar Typhimurium (Shao, Lei, et al., 2017). The re‐assembly of TJ structure induced by extracellular calcium repletion following calcium depletion is promoted by GPR39‐phospholipase C‐calcium/calmodulin‐dependent protein kinase kinase β‐AMPK pathways in T84 cells (Pongkorpsakol, Buasakdi, Chantivas, Chatsudthipong, & Muanprasat, 2019). In addition, zinc supplementation increases ZO‐1 expression via the phosphoinositide 3‐kinase/Akt/mammalian target of rapamycin pathway, resulting in the reinforcement of the TJ barrier in Caco‐2 cells (Shao, Wolf, et al., 2017). Furthermore, intracellular zinc in the intestinal epithelial cells plays an essential role in the maintenance of the TJ barrier. Depletion of intracellular zinc induces occludin proteolysis and downregulates claudin‐3 gene transcription, resulting in the disruption of the TJ barrier (Miyoshi, Tanabe, & Suzuki, 2016).

## 6 VITAMINS

Vitamins are a group of organic compounds that are essential in small quantities for normal growth and nutrition. Fat‐soluble vitamins A and D, and water‐soluble vitamin C reportedly play roles in the regulation of the intestinal barrier.

### 6.1 Vitamin A

Dietary restriction of vitamin A for 4 weeks in rats impairs the architecture and TJ barrier in the small intestine, as indicated by villi damage and reduced levels of TJ proteins, such as ZO‐1, occludin, and claudin‐1 (Xiao et al., 2019). These abnormalities are reversed by vitamin A supplementation for 15 d. Supplementation of all‐*trans* retinoic acid, an active metabolite of vitamin A, upregulates ZO‐1 and ZO‐2 levels in human intestinal organoids and Caco‐2 cells, respectively (Li et al., 2017; Yamada & Kanda, 2019). Furthermore, vitamin A protects the intestinal TJ barrier. In a murine model of necrotizing enterocolitis, administration of vitamin A reduces the inflammation by protecting the intestinal barrier (Xiao et al., 2018). Specifically, vitamin A administration increases the expression of ZO‐1, occludin, and claudin‐1 in a mouse model of necrotizing enterocolitis. Similarly, vitamin A reduces the TER and TJ protein levels induced by LPS and *Clostridium difficile* toxin A in Caco‐2 cells (Maciel et al., 2007; Xiao et al., 2018). The protective effect of vitamin A against LPS toxicity is also observed in porcine IPEC‐J2 cells (He et al., 2019).

### 6.2 Vitamin D

Similarly, vitamin D plays important roles in the maintenance and protection of the intestinal TJ barrier. Administration of vitamin D reduces the impairment of the TJ barrier in murine models of colitis, celiac disease, cirrhosis, and severe burn (Dong, Singh, Wei, Yao, & Wang, 2018; Liu et al., 2016; Stio, Retico, Annese, & Bonanomi, 2016; Wang, Yao, Hu, & Li, 2019). Dietary restriction of vitamin D exacerbates the TJ barrier defect in the *Citrobacter rodentium*‐induced colitic mouse (Assa et al., 2015). In inflamed tissues of individuals with ulcerative colitis, upregulation of claudin‐1 and claudin‐2 levels, and downregulation of claudin‐4 and claudin‐7 levels are observed, and the treatment of tissue biopsies with vitamin D corrects these abnormalities (Stio et al., 2016).

Vitamin D often exerts biological functions by acting via a vitamin D receptor (VDR), which belongs to the nuclear receptor superfamily of steroid/thyroid hormone receptors. VDR is expressed in most organs, including the intestinal epithelium, and transcriptionally regulates gene expression. Although no obvious defect in the TJ barrier, except for the downregulation of claudin‐2 levels, is apparent in the *Vdr* KO mouse, the KO mouse is more susceptible to DSS‐ and trinitrobenzene‐induced experimental colitis than a wild‐type mouse (Du et al., 2015; Kong et al., 2008; Kuhne et al., 2016). In the colitic model, intestinal hyperpermeability and reduction in TJ protein levels in the colon of the *Vdr* KO mouse are apparent at an earlier stage than those in the wild‐type mouse. Furthermore, in intestinal SW480 cells, vitamin D treatment increases ZO‐1, claudin‐1, and claudin‐2 levels, whereas *Vdr* knockdown compromises the increase in ZO‐1 levels (Kong et al., 2008). In addition, vitamin D reduces the MLCK expression and hyperpermeability induced by TNF‐α in a VDR‐dependent fashion (Chen et al., 2015; Du et al., 2015).

### 6.3 Vitamin C

Oral administration of ascorbic acid (vitamin C) increases the intestinal permeability to lactulose in healthy females, although the underlying mechanism remains unknown (Sequeira, Kruger, Hurst, & Lentle, 2015).

## 7 DIETARY FIBER (DF)

Dietary fibers are defined as carbohydrates that are not digested in the small intestine and hence reach the large intestine. DF intake exerts different physiological and biological effects on human health, depending on their molecular structure and physicochemical properties. Regulation of the intestinal barrier by DFs has been examined, with a focus on gut microbial metabolism. Fermentable DFs are easily metabolized by intestinal microorganisms and often alter the composition of the microflora and metabolites. In this section, I describe the effects of major microbial DF metabolites, SCFAs, on the intestinal TJ barrier.

The major SCFAs, acetate, propionate, and butyrate, play important roles in the maintenance, promotion, and protection of the intestinal TJ barrier. SCFA mixtures, whose compositions are relevant to the luminal environment, increase the TER and decrease the Lucifer yellow permeability of cecal mucosa in rats, indicating an enhanced integrity of the TJ barrier (Suzuki, Yoshida, & Hara, 2008). Similar observations were made with intestinal Caco‐2 and T84 cells.

Different mechanisms underlie the promotive effect of butyrate on the TJ barrier. Kelly et al. (2015) demonstrated that butyrate stimulates the epithelial metabolism and depletes intracellular oxygen, resulting in the stabilization of the transcription factor HIF‐1 and enhanced barrier integrity. Yan and Ajuwon (2017) and Feng et al. (2018) demonstrated that butyrate induces claudin‐3 expression via the Akt pathway in the colon of weaned piglets, porcine intestinal IPEC‐J2, and human intestinal Caco‐2 cells. Ohata, Usami, and Miyoshi (2005) suggested that butyrate increases lipoxygenase expression and TJ barrier integrity via cellular production of hydroxyeicosatetraenoic acid in Caco‐2 cells. In addition, butyrate reportedly promotes the formation of the TJ barrier. The re‐assembly of TJ structure induced by calcium resupply after calcium depletion is accelerated by butyrate via activation of PKCβ and AMPK, as well as reduction in MLC phosphorylation in Caco‐2 cells (Miao et al., 2016; Peng, Li, Green, Holzman, & Lin, 2009). The effects of acetate and propionate on the regulation of TJ barrier were also examined, but the evidence to date is scarce in comparison with that available for butyrate and the underlying mechanisms remain unknown.

## 8 POLYPHENOLS

Polyphenols are a heterogeneous group of compounds containing benzene rings with two or more hydroxyl groups (‐OH) attached. Different polyphenols may potentially regulate the integrity and structure of the TJ barrier, although the underlying mechanisms are still under investigation. Some polyphenols regulate the TJ structure by affecting the expression and assembly of the TJ‐associated proteins. In this section, I will focus on the effects of quercetin, naringenin, kaempferol, and resveratrol on the intestinal TJ barrier.

### 8.1 Quercetin

Quercetin belongs to the flavonol subgroup in the flavonoid group of compounds. High levels of this compound are present in onion, kale, and apple. Quercetin reportedly exerts several health promoting effects, such as anti‐carcinogenic and anti‐oxidative effects. Quercetin enhances the integrity of the intestinal TJ barrier by different mechanisms. One of the major mechanisms involves the upregulation of claudin‐4 levels (Amasheh et al., 2008; Suzuki & Hara, 2009). Luciferase reporter assays using claudin‐4 promoter plasmids revealed that transcription factors SP1, AP1, and GATA are involved in the quercetin‐mediated transcriptional regulation of claudin‐4 expression (Noda, Tanabe, & Suzuki, 2014). Not only intact quercetin, but also its two decomposition products, 3,4‐dihydroxybenzoic acid and 2,4,6,‐trihydroxybenzoic acid, appear to be able to upregulate claudin‐4 expression (Amasheh, Andres, Amasheh, Fromm, & Schulzke, 2009). In addition, non‐transcriptional regulation of TJ protein levels is observed earlier than the transcriptional regulation of claudin‐4 in Caco‐2 cells (Suzuki & Hara, 2009).

Quercetin also promotes the assembly of TJ proteins, ZO‐2, occludin, and claudin‐1 in the TJ structure of Caco‐2 cells (Suzuki & Hara, 2009). Amasheh et al. (2012) demonstrated that quercetin restores TNF‐α–induced TJ permeability at least in part by downregulating claudin‐2 levels. In a DSS‐induced experimental colitic mouse model, severe barrier loss is apparent, as indicated by decreased occludin, claudin‐3, and claudin‐4 levels in the colon. However, quercetin supplementation restores occludin and claudin‐3 expression, and reduces the inflammation (Shigeshiro, Tanabe, & Suzuki, 2013).

### 8.2 Naringenin

Naringenin belongs to the flavanone subgroup in the flavonoid group of compounds. It is present in high levels in citrus fruits, such as grapefruit. Naringenin supplementation preserves the colonic TJ structure, as indicated by increased occludin, JAM‐A, and claudin‐3 levels, in the colon of DSS‐induced colitic mice (Azuma, Shigeshiro, Kodama, Tanabe, & Suzuki, 2013). In Caco‐2 cells, naringenin promotes the assembly of ZO‐2, occludin, and claudin‐1 into the TJ structure (Noda, Tanabe, & Suzuki, 2013). Increased phosphorylation of occludin is at least in part related to naringenin‐mediated TJ assembly. Several lines of evidence indicate that occludin phosphorylation on Thr and Ser residues is essential for the assembly and maintenance of TJ structure (Manda et al., 2018; Suzuki et al., 2009). In addition, naringenin results in transcriptional upregulation of claudin‐4 expression through the SP1 pathway.

### 8.3 Kaempferol

Kaempferol is a flavonol compound. It is present in high levels in broccoli, chives, and kale. Kaempferol promotes the assembly of some TJ proteins, such as ZO‐1, ZO‐2, occludin, claudin‐1, claudin‐3, and claudin‐4, into the TJ structure. Cellular expression of ZO‐2 and claudin‐4 is also upregulated by kaempferol (Suzuki, Tanabe, & Hara, 2011). The precise mechanisms underlying kaempferol‐mediated TJ assembly remain unclear. However, TJ assembly is impaired upon the depletion of membrane cholesterol, which disturbs the structure and function of lipid microdomains in the plasma membrane of Caco‐2 cells. Accumulating evidence indicates that lipid microdomains, such as caveolae and rafts, are formed when a considerable amount of cholesterol is clustered with sphingomyelin and glycosphingolipids. Moreover, they sequester a variety of membrane proteins, including signaling molecules. These microdomains act as platforms for various signaling pathways, leading to the regulation of cellular function (Helms & Zurzolo, 2004). Interaction of the TJ protein complex with lipid microdomains has been demonstrated (Lambert, O'Neill, & Padfield, 2007; Nusrat et al., 2000). These findings suggest that the interaction of kaempferol with lipid microdomains or associated proteins plays a role in the promotion of TJ barrier integrity.

### 8.4 Resveratrol

Resveratrol belongs to the stilbenoid group of compounds. It is abundant in plants and plant products, such as grape, peanut, and red wine. Resveratrol supplementation restores the expression of TJ proteins, such as ZO‐2, occludin, JAM‐A, claudin‐3, claudin‐4, and claudin‐7, and mitigates the increased levels of plasma lipopolysaccharide‐binding protein, an indicator of intestinal barrier impairment in the DSS‐induced colitic mouse model (Mayangsari & Suzuki, 2018a). DSS‐induced infiltration of neutrophils and tissue destruction are mitigated by resveratrol in colitic mice. Resveratrol also alleviates the heat stress‐induced impairment of the TJ barrier in broiler chickens. Cyclic heat stress (10 hr/day for 3 weeks) increases the intestinal permeability to FITC‐labeled dextran with an accompanying reduction in ZO‐1, occludin, and claudin‐1 expression in the jejunum of broiler (Zhang et al., 2017). By contrast, resveratrol supplementation alleviates intestinal hyperpermeability and occludin and claudin‐1 expression. In vitro studies using Caco‐2 cells revealed that resveratrol exhibits promotive and protective effects on the TJ barrier (Mayangsari & Suzuki, 2018b). Resveratrol increases the expression of ZO‐2, occludin, and JAM‐A, and promotes the assembly of ZO‐1, ZO‐2, occludin, claudin‐1, claudin‐3, and claudin‐4 into the TJ structure. In addition, resveratrol reduces the hyperpermeability of TJ pathways induced by oxidative stress and IL‐6 (Mayangsari & Suzuki, 2018b). Resveratrol also mitigates the oxidative stress‐induced reduction in occludin expression in Caco‐2 cells. IL‐6 induces intestinal hyperpermeability by upregulating the pore‐forming claudin‐2 through the ERK1/2 and phosphoinositide 3‐kinase pathways (Suzuki, Yoshinaga, et al., 2011). Resveratrol suppresses IL‐6–induced ERK1/2 activation and thereby decreases claudin‐2 expression.

### 8.5 Other polyphenols

Some other polyphenols, in addition to the ones described in the preceding subsections, reportedly regulate the structure and integrity of the intestinal TJ barrier. The author of the current review and colleagues used Caco‐2 cells to demonstrate that daidzein, hesperetin, morin, and myricetin enhance the integrity of the TJ barrier, whereas chrysin decreases TJ integrity (Noda, Tanabe, & Suzuki, 2012; Suzuki & Hara, 2009). Furthermore, curcumin, genistein, and epigallocatechin gallate protect the TJ barrier against harmful stimuli, such as oxidative stress, inflammatory cytokines, infection, and acetaldehyde (Al‐Sadi & Ma, 2007; Atkinson & Rao, 2001; Lobo de Sa et al., 2019; Rao, Basuroy, Rao, Karnaky, & Gupta, 2002; Watson et al., 2004; Ye, Ma, & Ma, 2006).

## 9 CONCLUSION

Basic and clinical studies indicate the occurrence of intestinal hyperpermeability and impaired TJ structure in intestinal and extra‐intestinal disorders. The promotion and protection of the intestinal barrier by food factors and nutrition could be beneficial for general health. However, further studies should be performed to address some issues, such as the precise mechanisms underlying the regulation of the intestinal barrier by dietary factors, which remain unclear. Furthermore, in vivo studies, including clinical studies, are relatively scarce in many cases. Conducting these will provide information supporting the health‐promoting therapeutic potential of intestinal barrier regulation by nutritional components.
